# Stroke as an Initial Presentation of Hepatocellular Carcinoma: A Case Report

**DOI:** 10.1002/ccr3.71767

**Published:** 2026-01-02

**Authors:** Fereydun Moradi, Jamshid Tabeshpour, Kaveh Bahrami

**Affiliations:** ^1^ Department of Neurology Gonabad University of Medical Sciences Gonabad Iran; ^2^ Department of Pharmacy, Da.C. Islamic Azad University Damghan Iran; ^3^ Department of Neurology Mashhad University of Medical Sciences Mashhad Iran

**Keywords:** cryptogenic stroke, hepatocellular carcinoma, ischemic stroke, malignancy, paraneoplastic syndrome, stroke of undetermined source

## Abstract

Malignancy and ischemic stroke are major causes of global morbidity and mortality. Thromboembolic strokes secondary to malignancy are uncommon but represent a critical consideration in cryptogenic stroke. We report the case of a 46‐year‐old female with no significant past medical history who presented with acute rightward gaze deviation. Brain MRI confirmed acute multifocal ischemic infarcts. An extensive workup, including CT angiography and echocardiography, ruled out common stroke mechanisms, leading to a diagnosis of embolic stroke of undetermined source (ESUS) per 2021 AHA guidelines. This prompted a hypercoagulability workup, which revealed a markedly elevated D‐dimer. Subsequent investigation showed elevated liver enzymes, and abdominal imaging identified a hepatic mass with contrast‐enhanced CT features highly suggestive of hepatocellular carcinoma (HCC). The patient was transferred to oncology services for HCC management. At discharge, her neurological symptoms had partially improved, but she was lost to long‐term follow‐up, and thus her final oncological outcome is unknown.

## Key Clinical Message

Stroke can be the initial manifestation of occult hepatocellular carcinoma. Unexplained elevated D‐dimer and multifocal infarcts should prompt malignancy screening, including abdominal imaging, even in patients without typical cancer symptoms or liver test abnormalities. Early detection may guide appropriate oncologic and stroke management.

## 1 Introduction

Thromboembolic strokes secondary to malignancy are uncommon. However, both malignancy and ischemic stroke represent significant contributors to global morbidity and mortality [1, 2]. Among patients with cryptogenic stroke, an underlying malignancy is identified in approximately 5%–10% of cases, presenting a considerable diagnostic and therapeutic challenge. Cryptogenic stroke, defined as ischemic stroke without an identifiable etiology despite comprehensive evaluation, encompasses diverse pathophysiological mechanisms, broadly classified as embolic and non‐embolic. Embolic etiologies include malignancy and occult paroxysmal atrial fibrillation, among others.

The embolic stroke of undetermined source (ESUS) framework was developed to classify a significant subset of cryptogenic strokes—accounting for up to 20%–40% of ischemic strokes—and to guide the search for specific embolic causes [3]. Among these, occult malignancy is a critical, though often overlooked, etiology. Cancer can promote a hypercoagulable state through various mechanisms, including the production of procoagulant factors (e.g., tissue factor), leading to systemic thrombosis and embolism. Hepatocellular carcinoma (HCC), in particular, is associated with a significant risk of thromboembolic events. The reported incidence of stroke in HCC patients varies but is a well‐recognized complication, contributing to the morbidity of the disease. This hypercoagulable state is identified as the underlying cause in a substantial proportion (approximately 5%–10%) of cryptogenic strokes [1, 2]. Consequently, cerebrovascular disease represents the second most frequent neurological complication in cancer patients, following metastatic disease [4]. This case underscores the importance of considering an occult malignancy, such as hepatocellular carcinoma, in the differential diagnosis of patients presenting with ESUS, particularly when suggestive laboratory markers like elevated D‐dimer are present.

## 2 Case History

A 46‐year‐old woman presented to the neurology emergency department within 3 h of a sudden‐onset fall. She had no significant past medical history of hypertension, diabetes, or hyperlipidemia. She denied any history of smoking, alcohol use, or intravenous drug use. There was no personal or family history of liver disease, hypercoagulability, or stroke. The fall was not a mechanical trip but rather a sudden loss of postural control, suggesting a drop attack. This was accompanied by a forced, involuntary rightward gaze deviation. She also reported associated nausea and vertigo, but no headache or focal weakness. On initial assessment, her National Institutes of Health Stroke Scale (NIHSS) score was 3, driven solely by the conjugate eye deviation (score of 2) and mild ataxia (score of 1) noted on the coordination exam due to vertigo.

On neurological examination, the patient was alert and oriented. The key finding was a persistent rightward gaze preference, which could be overcome with the oculocephalic maneuver, confirming a cortical origin. Cranial nerve examination was otherwise unremarkable. Motor and sensory examinations revealed no focal weakness or sensory deficits. Coordination testing was limited by vertigo.

An urgent brain MRI was performed, which revealed diffusion restriction on DWI with corresponding ADC hypointensity and FLAIR hyperintensity in the bilateral cerebellar hemispheres, left parieto‐occipital, and right parieto‐frontal regions, confirming acute multifocal infarcts in multiple vascular territories.

An urgent brain MRI was performed, which revealed diffusion restriction on DWI with corresponding ADC hypointensity and FLAIR hyperintensity in the bilateral cerebellar hemispheres, left parieto‐occipital, and right parieto‐frontal regions, confirming acute multifocal infarcts in the posterior and anterior circulation territories. Brain and neck CT angiography demonstrated only moderate stenosis (< 50%) in the V4 segment of the vertebral artery, which was deemed insufficient to cause the multifocal infarcts. Transthoracic echocardiography was unremarkable, with no evidence of a cardioembolic source. A 30‐day outpatient cardiac event monitor showed no paroxysmal atrial fibrillation. A subsequent transesophageal echocardiogram (TEE) with agitated saline (bubble) study was performed. The TEE revealed no evidence of an intracardiac thrombus, valvular vegetation, or aortic arch atheroma. The bubble study, performed at rest and with Valsalva maneuver, was negative, definitively ruling out a patent foramen ovale (PFO) or any other right‐to‐left shunt. With all other major embolic sources excluded, the diagnosis was classified as an ESUS. Given this ESUS classification and the high clinical suspicion for a hypercoagulable state, further laboratory investigations were pursued

**FIGURE 1:**
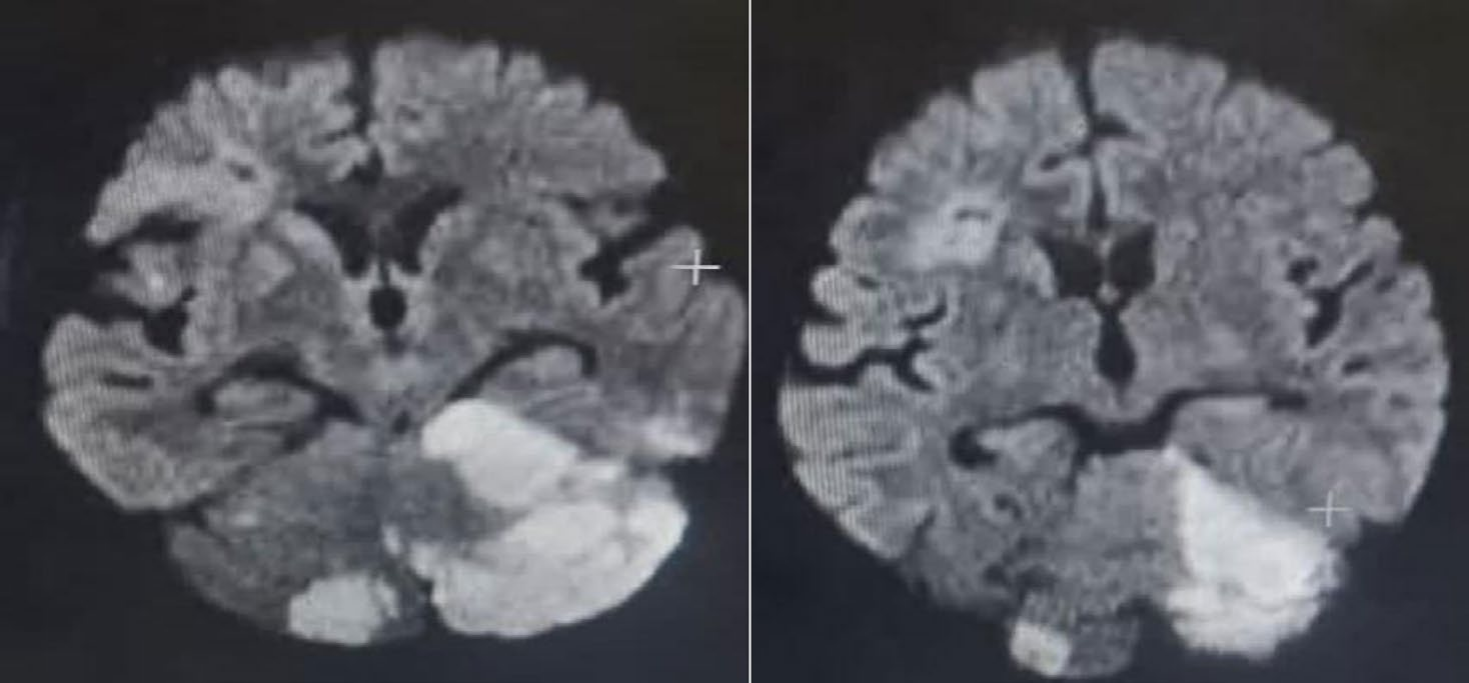
**— Contrast‐enhanced** abdominal CT scan of the liver demonstrating a large, mass‐like lesion (50 × 45 mm) in the right hepatic lobe (arrow), with imaging characteristics highly suggestive of hepatocellular carcinoma.

This unexplained hypercoagulability prompted a systemic evaluation for occult malignancy. Subsequent laboratory studies revealed a normal complete blood count, basic metabolic panel, and coagulation profile (PT/INR, aPTT). However, hypercoagulability was indicated by a plasma D‐dimer level, measured within 48 h of symptom onset, elevated at five times the upper limit of normal (2.5 mg/L FEU, reference < 0.5). Furthermore, liver function tests were elevated (AST 120 U/L, ALT 145 U/L, ALP 220 U/L) with associated hyperbilirubinemia (Total 2.1 mg/dL, Direct 1.5 mg/dL). Notably, serum alpha‐fetoprotein (AFP) was not measured during the initial stroke workup. An abdominal ultrasound was subsequently performed, which identified multiple hypoechoic foci with interspersed hyperechoic areas and poorly defined borders in the right hepatic lobe, the largest measuring 50 × 45 mm. A contrast‐enhanced abdominal CT confirmed mass‐like lesions with characteristic arterial enhancement and washout, highly suggestive of HCC (Figure 1).

Although the contrast‐enhanced CT findings were highly characteristic of HCC according to standardized imaging criteria (LI‐RADS 5), a tissue biopsy was not performed prior to initiating oncology care. Serum alpha‐fetoprotein and hepatitis serology were not obtained during the initial stroke workup. As noted, serum alpha‐fetoprotein and hepatitis serology were not obtained during the initial stroke workup. The patient was promptly referred to hepatobiliary surgery and oncology for further management, where the clinical and radiological diagnosis of HCC was confirmed. An abdominal ultrasound and contrast‐enhanced CT were performed primarily for lesion characterization. The radiology reports described the hepatic mass in detail but did not include a specific assessment of the background liver parenchyma for features of cirrhosis. Therefore, the presence or absence of underlying cirrhosis in this patient remains undetermined.

The patient's acute ischemic stroke was managed with antiplatelet therapy (Aspirin 100 mg daily) and high‐dose statin (Atorvastatin 40 mg daily). Following the diagnosis of HCC, she was transferred to the oncology and hepatobiliary surgery services for further management. At the time of discharge, her neurological symptoms, including the gaze deviation and vertigo, had partially improved with supportive care.

## 3 Conclusion and Results

This case highlights that acute multifocal ischemic stroke can be the initial manifestation of an occult hepatocellular carcinoma. In patients with an ESUS, a significantly elevated D‐dimer level should prompt a systematic search for an underlying malignancy, including contrast‐enhanced abdominal imaging, even in the absence of typical cancer‐related symptoms or overt liver dysfunction. Incorporating this approach into the ESUS diagnostic algorithm is crucial for the early detection of occult cancers and guides appropriate, combined management of both the stroke and the malignancy.

## 4 Follow‐Up

The patient was discharged from the neurology service with a diagnosis of acute multifocal ischemic stroke secondary to occult hepatocellular carcinoma. She was transferred to the care of the oncology and hepatobiliary surgery services for further management of the HCC. Unfortunately, the patient was lost to long‐term follow‐up thereafter. Consequently, no data are available regarding the specific oncological treatment she received, her response to that treatment, the potential recurrence of stroke, or her final clinical outcome.

## 5 Discussion

The patient presented with multifocal ischemic infarcts affecting multiple vascular territories, with laboratory investigations revealing an isolated elevation in D‐dimer levels. We acknowledge the important limitation that D‐dimer is an acute‐phase reactant and can be elevated in acute ischemic stroke irrespective of the etiology, as noted by the reviewer. The level in our patient was measured within 48 h of symptom onset. However, prior research has demonstrated that the degree of D‐dimer elevation is crucial; levels greater than five times the upper limit of normal, particularly in conjunction with a pattern of multiple embolic infarcts, have a much higher predictive value for occult malignancy than minor elevations seen in typical atherosclerotic or cardioembolic strokes [5]. Therefore, while the D‐dimer elevation in our patient was non‐specific, its marked magnitude in the context of a cryptogenic, multi‐territorial stroke was the pivotal finding that heightened our suspicion for a cancer‐related hypercoagulable state and justified the subsequent malignancy workup.

This case underscores the importance of a systematic and exhaustive evaluation for ESUS. In our patient, the multifocal infarcts prompted a comprehensive workup that included extended cardiac monitoring and TEE to confidently exclude occult paroxysmal atrial fibrillation and a right‐to‐left shunt, respectively. It was only after these essential investigations returned negative that the significantly elevated D‐dimer level became the pivotal clue pointing towards a cancer‐related hypercoagulable state as the underlying mechanism.

The pathophysiological link between HCC and ischemic stroke is multifactorial and, in this case, remains speculative. While our workup confidently excluded other major embolic sources, we did not find direct evidence for a specific mechanism in this patient. Specifically, the abdominal CT showed no evidence of portal vein thrombosis, and the transesophageal echocardiogram revealed no valvular vegetations to suggest non‐bacterial thrombotic endocarditis (NBTE). Furthermore, the bubble study confirmed the absence of a patent foramen ovale, effectively ruling out paradoxical embolism as a pathway in this instance.

Nevertheless, the literature describes several plausible mechanisms by which HCC can cause stroke. HCC is known to produce procoagulant factors and can trigger a systemic hypercoagulable state, which is the most likely overarching mechanism in our patient, as reflected by the markedly elevated D‐dimer. Other reported, though unconfirmed in our case, mechanisms include direct tumor invasion leading to portal vein thrombosis (with paradoxical embolism requiring a PFO), NBTE, or even rare tumor cell embolism. Therefore, while we can only confirm a temporal association between the stroke and the diagnosis of HCC, the exclusion of other causes and the presence of a known cancer‐related hypercoagulable state make a causal link highly plausible. This case underscores that HCC should be considered a potential source of embolic stroke, even in the absence of direct mechanistic proof or overt liver dysfunction.

The OASIS‐Cancer study further supports this association, reporting that cancer‐related ESUS patients exhibit significantly higher D‐dimer levels compared to non‐cancer ESUS controls [6]. Given this evidence, an extensive malignancy workup is warranted in cryptogenic stroke cases with unexplained hypercoagulability. Recommended screening modalities include contrast‐enhanced chest and abdominal CT, gastrointestinal endoscopy, and positron emission tomography (PET‐CT), as adenocarcinoma represents the most frequently identified malignancy in cancer‐associated ischemic stroke [7].

Notably, stroke may occur at any stage of malignancy and can even serve as the initial manifestation of an occult cancer in up to 3% of cases [8]. This case highlights that stroke may precede typical clinical or biochemical signs of HCC, underscoring the importance of malignancy screening even in the absence of hepatic symptoms or abnormal liver function tests. Therefore, in cases of cryptogenic stroke, particularly those with an ESUS pattern and elevated D‐dimer, screening for occult malignancy with contrast‐enhanced CT of the chest and abdomen should be considered a standard part of the diagnostic algorithm, even in patients without constitutional symptoms or classic laboratory abnormalities.

Furthermore, cancer patients remain at high risk for recurrent stroke, yet optimal secondary prevention strategies remain uncertain. The current literature highlights ongoing debates regarding the choice between antiplatelets, therapeutic anticoagulation, or direct oral anticoagulants for secondary prevention in cancer‐associated stroke, with no clear consensus established in guidelines [9, 10].

This case provides a clear clinical implication for the management of ESUS: an unexplained, significant elevation in D‐dimer (> 5× ULN) should be considered a major red flag for occult malignancy. This finding, especially in the context of multifocal infarcts, warrants an aggressive and systematic search for cancer, even in patients without typical oncological symptoms or overt laboratory abnormalities. We propose that contrast‐enhanced CT of the chest and abdomen should be a standard component of the ESUS workup in such scenarios, as it can efficiently identify occult solid tumors like HCC.

A limitation of this case is the lack of histopathological confirmation of the hepatocellular carcinoma. The diagnosis was based on characteristic imaging findings on contrast‐enhanced CT, which, in the appropriate clinical context, is considered diagnostic for HCC by major guidelines (such as the American Association for the Study of Liver Diseases, AASLD), thereby often obviating the need for biopsy, especially when the lesion is > 1–2 cm in a cirrhotic liver or shows classic vascular patterns (arterial phase hyperenhancement and washout) [11]. In our patient, the multifocal stroke provided the clinical impetus for an extensive workup that serendipitously revealed the liver mass. The decision to forgo biopsy was made by the managing oncology team in favor of expedited treatment planning. Future cases would be strengthened by the routine inclusion of AFP and hepatitis serology in the diagnostic workup for cancer‐associated stroke.

Another limitation in our case is the lack of a formal assessment for underlying cirrhosis, as the imaging was focused on characterizing the focal liver lesion. This highlights a crucial learning point: when a liver mass is discovered in the context of cryptogenic stroke, the radiologist should be specifically asked to comment on the background liver morphology. Furthermore, a more comprehensive evaluation for liver disease etiology, such as testing for non‐alcoholic fatty liver disease (NAFLD) or metabolic syndrome, could provide further context. The fact that our patient lacked traditional risk factors like viral hepatitis or significant alcohol history, yet still developed HCC, underscores that malignancy can occur even in non‐cirrhotic livers or in the context of subclinical liver disease. Future evaluations for cancer‐associated stroke should include a deliberate assessment for cirrhosis.

## Author Contributions

**Fereydun Moradi:** methodology, writing – original draft. **Jamshid Tabeshpour:** writing – review and editing. **Kaveh Bahrami:** conceptualization, supervision.

## Funding

The authors have nothing to report.

## Consent

Written informed consent was obtained from the patient to publish this report in accordance with the journal's patient consent policy.

## Conflicts of Interest

The authors declare no conflicts of interest.

## Data Availability

Data sharing not applicable to this article as no datasets were generated or analysed during the current study.
